# Multiple Sensor Synchronization with theRealSense RGB-D Camera

**DOI:** 10.3390/s21186276

**Published:** 2021-09-18

**Authors:** Hyunse Yoon, Mingyu Jang, Jungwoo Huh, Jiwoo Kang, Sanghoon Lee

**Affiliations:** 1 Department of Electrical and Electronic Engineering, Yonsei University, Seoul 03722, Korea; hsyoon97@yonsei.ac.kr (H.Y.); jmg1002@yonsei.ac.kr (M.J.); gjwjddn9@yonsei.ac.kr (J.H.); slee@yonsei.ac.kr (S.L.); 2 Department of Radiology, College of Medicine, Yonsei University, Seoul 03722, Korea

**Keywords:** RGB-D camera, 3D reconstruction, camera synchronization, timestamp, synchronization delay

## Abstract

When reconstructing a 3D object, it is difficult to obtain accurate 3D geometric information using a single camera. In order to capture detailed geometric information of a 3D object, it is inevitable to increase the number of cameras to capture the object. However, cameras need to be synchronized in order to simultaneously capture frames. If cameras are incorrectly synchronized, many artifacts are produced in the reconstructed 3D object. The RealSense RGB-D camera, which is commonly used for obtaining geometric information of a 3D object, provides synchronization modes to mitigate synchronization errors. However, the synchronization modes provided by theRealSense cameras can only sync depth cameras and have limitations in the number of cameras that can be synchronized using a single host due to the hardware issue of stable data transmission. Therefore, in this paper, we propose a novel synchronization method that synchronizes an arbitrary number of RealSense cameras by adjusting the number of hosts to support stable data transmission. Our method establishes a master–slave architecture in order to synchronize the system clocks of the hosts. While synchronizing the system clocks, delays that resulted from the process of synchronization were estimated so that the difference between the system clocks could be minimized. Through synchronization of the system clocks, cameras connected to the different hosts can be synchronized based on the timestamp of the data received by the hosts. Thus, our method synchronizes theRealSense cameras to simultaneously capture accurate 3D information of an object at a constant frame rate without dropping it.

## 1. Introduction

In the fields of computer vision and graphics, many studies have attempted to reconstruct 3D objects from a single image [1,2,3,4,5]. Techniques of 3D reconstruction from a single image simplify the process and reduce the total computational cost. However, the quality of the reconstructed 3D object has been a ubiquitous problem. A single image cannot grasp the full geometric information of the object due to regions occluded from the camera’s field of view. With missing geometric information, these methods can only make inaccurate inferences of the object’s geometry. As a result, the reconstructed unseen surfaces tend to be either overly smooth or have severe artifacts.

In contrast to the single-image method, the utilization of multiple sensors enables capturing the full 3D geometry of an object [6,7,8,9]. By fusing the surfaces observed by multiple cameras, the 3D object can be accurately reconstructed with fewer artifacts. However, the performance of 3D reconstruction using multiple sensors is heavily influenced by the synchronization of these sensors.

**Figure 1:**
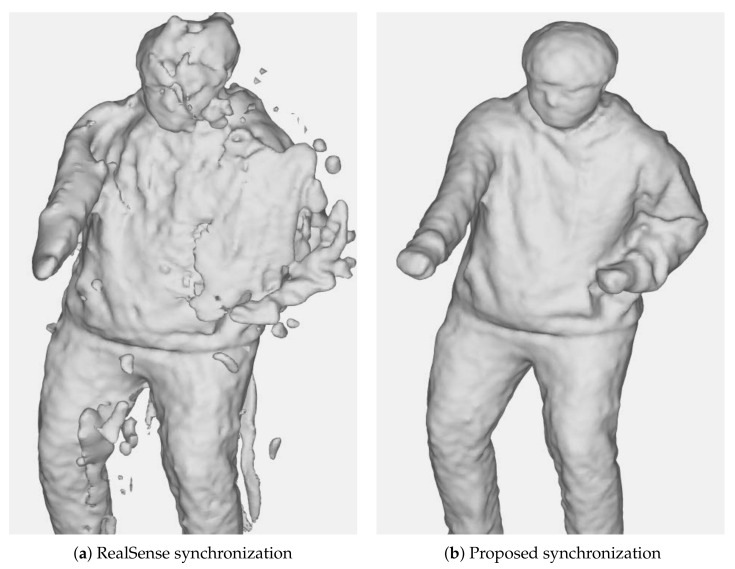
The reconstructions generated by a method in [9] using the data captured by 18 RGB-D cameras with (**a**) the Slave/Full Slave synchronization mode and (**b**) the proposed synchronization method, respectively.

For example, Figure 1 shows the case in which sensors in a multiview system are not correctly synchronized. In a situation in which an object is moving while the incorrectly synchronized sensors are temporally capturing an object, the object’s position in the world coordinates differs between sensors. Hence, the artifacts are generated when fusing these surface information. However, as shown in Figure 1b, if the sensors are synchronized, less artifacts are produced.

Intel’s RealSense RGB-D camera, known for its cost-efficiency and mobility, is widely used in 3D applications [10,11,12,13,14,15]. The RealSense RGB-D camera is supported by an open-source multiplatform SDK [16], librealsense, allowing simpler integration of the RealSense RGB-D camera. Furthermore, the RealSense RGB-D camera provides global shutter mode to alleviate synchronization issues and achieve better performance when capturing high-speed movement, avoiding depth image blurring or shooting in low-light situations [17,18]. The RealSense RGB-D camera provides three synchronization modes to mitigate synchronization issue: GenLock, Slave, and Full Slave.

GenLock mode is a function whereby multiple sensors synchronize upon the rising or falling edge of a signal generated from an external trigger. With this feature, depth frames can be triggered from an external source to capture at almost arbitrary times or frequencies, within an allowable time and frequency window. However, GenLock mode provided by the RealSense drops the frame rate of each sensor by half [19]. The reduction of the frame rate causes a decrease in the quality of temporal data and an increase in the vulnerability of data to motion blurs. In addition, RealSense’s mode currently only supports syncing for depth cameras, and RGB cameras cannot be synced using GenLock mode as they use a rolling shutter. Even though an RGB-D camera, such as the RealSense D455, consists of both global shutter RGB and depth cameras, GenLock mode cannot synchronize both cameras, because the necessary functionality for the synchronization of RGB and depth cameras has not been supported yet [19]. Due to the omission of RGB camera synchronization, reliable RGB data cannot be attained from a multiview camera system synchronized with GenLock.

Similar to GenLock mode, Slave mode only syncs the depth camera of RealSense cameras and syncs to a periodic signal generated from an external trigger. The difference between GenLock mode and Slave mode is that Slave mode allows cameras to start capturing frames without a trigger while GenLock only starts capturing frames when the signal is given. Moreover, unlike GenLock mode, which halves the frame rate, Slave mode enables the sensors to record at a designated frame rate. However, Slave mode faces a challenge in synchronizing multiple cameras. Due to the hardware issue of the limited USB bandwidth, the maximum number of devices that can be connected to a single host for stable data transmission is three [18]. In order to maintain the stability of data transmission, the number of hosts needs to increase to support a multiview RGB-D camera system. Although this constraint is applied to each synchronization mode, GenLock mode can still synchronize multiple sensors connected to different hosts because GenLock mode forces the sensors to synchronously capture frames at a designated time. In contrast to GenLock mode, Slave mode does not synchronize sensors at a designated time and, thus, synchronizes the sensors based on the host’s system clock in order to correct any delays affecting the order of the data packet transmission. Since all hosts have different system clocks, it is difficult for Slave mode to synchronize the sensors connected to different hosts. While Full Slave mode suffers the same issue as Slave mode, Full Slave mode is able to synchronize both the RGB camera and depth camera if both are global shutter cameras.

To tackle the problems faced by all these synchronization modes, we propose a novel synchronization method, which allows the synchronization of multiple RealSense cameras each connected to a different host. Because our method is an extension of Full Slave mode, our method can synchronize both RGB cameras and depth cameras in RealSense cameras and capture frames without dropping the frame rate. Furthermore, our method increases the number of synchronizable cameras by increasing the number of hosts in the multiview system so that more accurate geometric information of the 3D object can be obtained. Synchronization of multiple hosts was performed via setting all hosts to have the same system clock using the master–slave architecture by introducing two kinds of delays between the hosts: *time synchronization delay* and *network latency delay*. Having all hosts share a common system clock allows synchronization of RGB-D cameras connected to different hosts, allowing the reliable capture of numerous RealSense cameras simultaneously without a frame rate drop. In our experiments, 18 RealSense RGB-D cameras were used to validate the proposed method, demonstrating accurate and reliable synchronization of multiple RealSense cameras.

## 2. Related Work

### 2.1. RGB-D Camera

Many affordable RGB-D cameras are widely available today, accelerating research related to 3D reconstruction [20,21,22,23]. The most widely used commercially available cameras are the Orbbec Astra (Orbbec, Troy, MI, U.S.) series [24], the Microsoft Kinect Azure [25] (Microsoft, Redmond, WA, U.S.), and the Intel RealSense L515 [26] and Intel RealSense D400 series [18] (Intel, Santa Clara, CA, U.S.). Table 1 summarizes the specifications of these cameras.

The Orbbec Astra series uses a structured light technique to estimate depth. In the structured light technique, patterns whose original shapes are known in advance are projected to a target object. The depth is estimated using geometric relationships between the original and deformed pattern shapes. However, when the object is captured using multiple structured-light-based cameras, the patterns projected to the target object from different cameras interrupt each other. Thus, the deformed pattern shape from one camera cannot be distinguished clearly, making it difficult to use multiple structured-light-based cameras to capture a single scene.

The Microsoft Kinect Azure uses a time-of-flight (ToF) technique that estimates depth by measuring the round-trip time of light. The Kinect Azure device supports the highest RGB resolution among other comparison cameras. As a consequence of supporting the highest resolution, it requires an extra power supply in addition to the USB cable. Furthermore, as the ToF technique measures the depth using the signal reflected from the target object, the quality of the depth significantly decreases when multiple ToF cameras are used to capture the object simultaneously. The Intel RealSense L515 uses a light detection and ranging (LiDAR) technique, which is one of the ToF techniques. Although the depth can be estimated more accurately using the LiDAR technique compared to other techniques, it is difficult to apply multiple cameras to capture a single object simultaneously, similar to the structured light technique, as reported in [27].

The Intel RealSense D400 series uses an active stereo technique to estimate depth, where a projector emits an unstructured pattern to add texture to the surface of the object while the depth is calculated by matching texture correspondences between images captured by two infrared (IR) cameras. Among other cameras in Table 1, the Intel RealSense D400 series has a significant advantage in composing the multicamera system.

Firstly, it has been reported that the depth quality of a single camera can be improved using multiple active stereo cameras [19] since multiple projectors increase the resolution of the unstructured pattern projected on the target object and thus enable more accurate matching between the IR images. Therefore, employing multiple cameras is helpful to obtain better depths in contrast to other techniques, such as structured light, ToF, and LiDAR.

Secondly, the Intel RealSense D400 series provides a 90 FPS capture speed, whereas other cameras support up to 30 FPS. This enables a moving object to be captured more reliably and accurately. Finally, the Intel RealSense D400 series provides the highest depth resolution of 1280 × 720. This is consistent with the report [28] that the RealSense D400 series obtains the highest quality depth from a single view among recent depth cameras. With the notable advantages of the RealSense depth camera, we propose a reliable synchronization method for multiple RealSense cameras.

### 2.2. Time Synchronization

To synchronize multiple hosts in a system, it is important to set all hosts to have the same system clock. To synchronize the system clocks of all hosts, using the Network Time Protocol (NTP) is a standard procedure in many applications because of its high availability and its ease of use. The NTP synchronizes system clocks among a set of distributed time servers and clients over the Internet [29,30]. System clocks are synchronized by exchanging the timestamps between the time server and clients. While exchanging timestamps, the time taken for NTP data packets to complete a round-trip causes a delay in synchronization. In addition, a delay arises from an offset between the arrival and departure of the data packet from the client to the server. These delays are estimated to reduce the negative effects on synchronizing system clocks. However, when synchronizing the hosts in the same subnet of the network, the advantage of computing delays caused by the data packet’s round-trip is lost. The synchronization mode used for the hosts in the same subnet is called multicast mode. The NTP server in multicast mode periodically sends data packets to other hosts in the same subnet. The NTP clients receive the data packets and set their system clocks to the time stored in the data packets while assuming a few milliseconds of delay instead of actually computing it [31]. This is because the NTP server declines to accept any data packets from the clients and, thus, cannot compute the round-trip delays. As a result, when applying the NTP synchronization method to multiple hosts, the hosts are prone to have system clocks with a few milliseconds of delay. In order to tackle this challenge, we propose a reliable synchronization method for the hosts in the same subnet to have the same system clock.

## 3. Camera Synchronization Method

Because our method aims to accurately synchronize a multihost camera system, the timestamps measured by each sensor and host play significant roles in synchronizing the multihost camera system. The four timestamps provided from the camera are the *sensor*, *frame*, *backend*, and *time-of-arrival* timestamps. While both the *sensor* and *frame* timestamps are measured by the camera’s device clock, the *backend* and *time-of-arrival* timestamps are measured by the host’s system clock.

**Figure 2:**
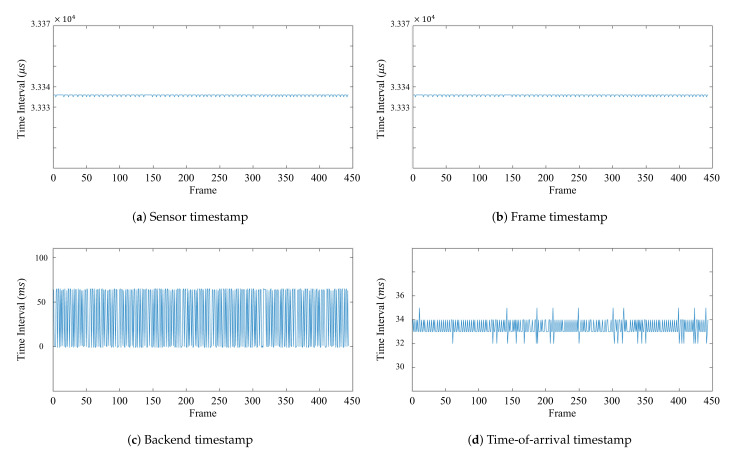
Intervals according to the frame of each timestamp.

Figure 2 illustrates the time intervals between consecutive frames of a sensor with regard to the four different timestamps. All intervals are the same, indicating that a specific number of FPS were captured at that point in time. The *sensor* timestamp is a timestamp that marks the middle of the camera exposure, and the *frame* timestamp specifies the time when the first packet is sent to a host. Figure 2a,b shows that the time interval between consecutive *sensor* and *frame* timestamps is almost consistent and that they are similar to each other. The *back-end* timestamp measures the time when the host copies the data packet from the USB controller to the OS kernel. From Figure 2c, one can observe that time interval between consecutive *back-end* timestamps drastically fluctuates. The *time-of-arrival* timestamp then measures the time when the RealSense SDK, librealsense, receives the data, and Figure 2d shows that the time interval between consecutive *time-of-arrival* timestamps does not fluctuate as much as in Figure 2c. However, there are some random big jumps in the middle, which shows that there are delays in data transmission between the RGB-D camera and the host.

**Figure 3:**
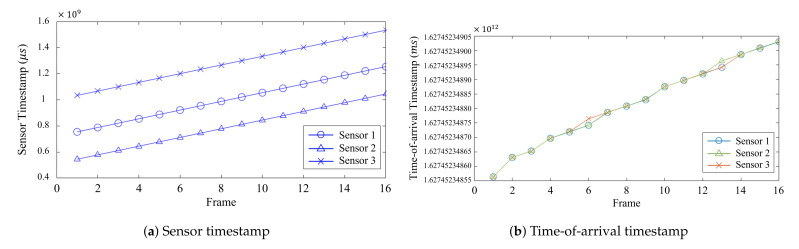
The *sensor* and *time-of-arrival* timestamps of each frame.

Figure 3 shows both the *sensor* and *time-of-arrival* timestamps of the three RealSense RGB-D cameras connected to the same host.Although the *sensor* timestamp has consistent intervals in Figure 3a, they are generated by the cameras’ clocks. This difference between the cameras’ *sensor* timestamps makes the synchronization difficult. On the other hand, the *time-of-arrival* timestamps of the three cameras, as illustrated in Figure 3b, almost match each other. This is because the *time-of-arrival* timestamp is measured by one host, which receives the data from the three cameras. Since the *time-of-arrival* timestamps match one another, it is easier to determine whether synchronization has been correctly performed or not. However, there are some sudden jumps shown in Figure 2d, causing the *time-of-arrival* timestamps of the cameras to not match at certain frames. Thus, the regressed linear model between the *sensor* and *time-of-arrival* timestamps, which is called the *global* timestamp, was used to correct big jumps in the delay, as shown in Figure 2d, and synchronize the RealSense RGB-D cameras.

### 3.1. Single Host Synchronization

The difference between the *sensor* or *time-of-arrival* timestamps of sequential frames should be consistent as the sensor captures frames at a fixed frame rate. However, in some cases, the frame can be occasionally captured outside the periodic frame rate. Thus, in order to correct these outliers, the linear regression between the *sensor* and *time-of-arrival* timestamps was modeled. Let $t_{s}$ and $t_{h}$ be the *sensor* and *time-of-arrival* timestamps. The regression of the *global* timestamp $t_{g}$ is defined as:

(1) $$\begin{array}{cc} t_{g} & =a\Delta t_{s}+b+t_{h},\\ a & =\frac{\sum_{i=1}^{n}\Delta t_{h}^{i}\sum_{i=1}^{n}{\left(\Delta t_{s}^{i}\right)}^{2}-\sum_{i=1}^{n}\Delta t_{s}^{i}\sum_{i=1}^{n}\Delta t_{s}^{i}\Delta t_{h}^{i}}{n\sum_{i=1}^{n}{\left(\Delta t_{s}^{i}\right)}^{2}-{\left(\sum_{i=1}^{n}\Delta t_{s}^{i}\right)}^{2}},\\ b & =\frac{n\sum_{i=1}^{n}\Delta t_{s}^{i}\Delta t_{h}^{i}-\sum_{i=1}^{n}\Delta t_{s}^{i}\sum_{i=1}^{n}\Delta t_{h}^{i}}{n\sum_{i=1}^{n}{\left(\Delta t_{s}^{i}\right)}^{2}-{\left(\sum_{i=1}^{n}\Delta t_{s}^{i}\right)}^{2}}, \end{array}$$

where Δ is the delta operator to calculate the time interval between the previous and current timestamps, *a* and *b* are the coefficients of the linear regression, and *n* is the number of the previous frame for the linear regression, respectively. RealSense cameras connected to the same host can be synchronized via the regression of the *global* timestamp. The application of synchronization can be categorized into two scenarios: online and offline. Hence, we computed the linear regression in (1) in different ways for each scenario.

In the online scenario, we regressed a linear model between the *sensor* and *time-of-arrival* timestamps while capturing scenes in real time. Coefficients *a* and *b* of the linear regression were estimated using the timestamps from the current frame to the previous *n* frame. Using the newly updated coefficients of each frame, a *global* timestamp $t_{g}$ was estimated. However, for the first *n* frames, which have less than *n* frames for a regression, frames from the starting point to the current frame were used to regress the global timestamps.

In the offline scenario, the *global* timestamps were regressed using full data captured after a recording was completed. Because regression in (1) was performed on full data, a single coefficient pair *a* and *b* was used to estimate the *global* timestamp of each frame.

**Figure 4:**
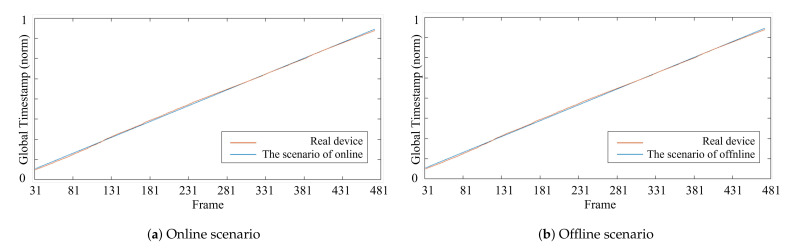
The *global* timestamps generated in (**a**) the online scenario and (**b**) the offline scenario.

Figure 4 shows the regressed global timestamps computed in the two different scenarios. Figure 4a,b shows the distribution of the *global* timestamps in the online and offline scenarios, respectively. In Figure 4a, the *global* timestamps were regressed based on the recent last frames (n=30) using the regression model in (1), and Figure 4b shows the linear regression on all frames (n=480) captured by a sensor. We can observe that there were no significant differences between the online and offline scenarios, as shown in Figure 4. However, because the method used to model Figure 4a can be applied in real time, using the online scenario in (1) to regress the *global* timestamps seemed more suitable when obtaining temporal RGB-D data in real time.

In an ideal condition, an arbitrary number of sensors can be connected to a single host. Under this ideal condition, all sensors can be synchronized via a global condition, and thus, a complete multiview sensor system can be established. However, in reality, the USB bandwidth of a host can only support up to three RealSense cameras for stable data transmission. With only three sensors, it is difficult to obtain accurate 3D geometric information for many application. Therefore, to increase the number of cameras, it is necessary to increase the number of hosts as well.

**Figure 5:**
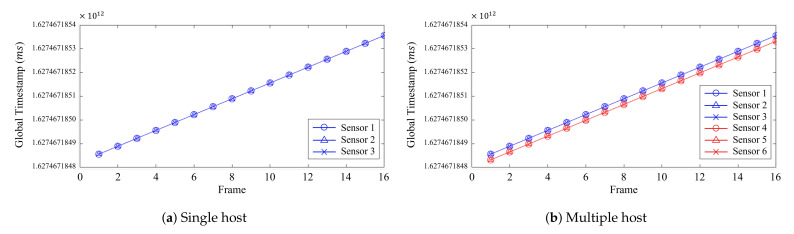
The *global* timestamps of the three cameras connected to (**a**) a single host and (**b**) six cameras connected to two hosts.

Figure 5 illustrates the result of the *global* timestamps attained from a single- and multiple-host system. Figure 5a illustrates the *global* timestamps of the three cameras connected to a single host, and Figure 5b illustrates the *global* timestamps of six cameras connected to two hosts in groups of three. Using the *global* timestamps, the synchronization of the three cameras in the single-host system seemed successful, as seen in Figure 5a. However, when using multiple hosts to expand to more devices, the *global* timestamps were created with similar values only for the cameras connected to the same host, as shown in Figure 5b. Thus, the *global* timestamps depended on the hosts. This difference in timestamps between hosts shows that the cameras connected to the same host could be synchronized, but could not be with other cameras connected to different hosts.

### 3.2. Multiple Host Synchronization

Our method synchronizes the sensors using the *global* timestamps, which is regressed from the *sensor* and *time-of-arrival* timestamps. The use of the *global* timestamps allows the synchronization of the sensors connected to the same host, but the application of the *global* timestamps to a multihost sensor system required some modification. This was accomplished by increasing the number of synchronized hosts, unlimiting the number of cameras to be captured simultaneously without a frame rate drop. To extend the regression of the global timestamps from a single host to multiple hosts, the system clock of each host needed to be synchronized first. Having all hosts share the same system clock was an important factor because a small difference in the hosts’ system clocks could cause considerable differences in the *global* timestamps across cameras. Not only did the difference between system clocks cause incorrect synchronization, but also, the delays in the process of system clock synchronization produced a significant error in the estimation of the *global* timestamps. Thus, our method estimates the delay in the system clock synchronization and the time difference to correctly synchronize the hosts’ system clocks and regress accurate *global* timestamps for multiple hosts. Since each host had a different system clock, one of the host was set as the master host, and the other hosts were set as the slave hosts.

After specifying the master host, each slave host’s system clock was set to match the master host’s system clock sequentially. The configuration of the slave’s system clock was performed by adding the total delay of the synchronization procedure, defined as:

(2) $$\begin{array}{c} t_{d}=t_{t}+t_{l} \end{array}$$

where $t_{d}$ is the total delay of the addition of *time synchronization delay* $t_{t}$ and *network synchronization delay* $t_{l}$. The *time synchronization delay* is the time taken for the slave host to receive the master host’s system clock and set it as its own. his was obtained by computing the average time for the host to call the *get-time* and *set-time* operating system functions $n_{c}$ times. The assumption for the time synchronization delay was that all hosts took the same amount of delay when calling the *get-time* and *set-time* operating system functions.

The *network synchronization delay* was measured by calculating the average time taken for the transmission of $n_{d}$ data packets between the master and slave hosts. The master host sends its system time to a slave host via network communication, and it takes time for the data to reach a slave host. By the time the slave host receives the data, the master’s system clock no longer matches that of the slave host. Thus, it is important to estimate the delay in the network in order to remove any difference between the master’s and slave’s system clocks. The synchronization procedure between the master and slave hosts was repeated until the synchronization delay was within the predefined threshold of the synchronization $T_{th}$.

Our method of setting the slave’s system clock allows establishing a multiview sensor system that is scalable and flexible because the number of hosts can easily be increased or decreased without affecting the other hosts. After setting the slave’s system clock to match that of the master’s, multiple hosts can be considered as a single host, as they share the same clock. Hence, the single-host synchronization method can be applied to multiple-host systems. The detailed procedures of the master and slave hosts are represented in Algorithms 1 and 2, respectively.
**Algorithm 1:** Operation at the master host.
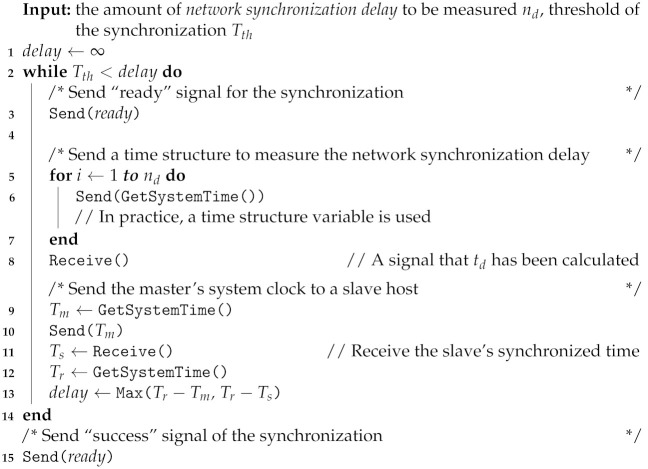




**Algorithm 2:** Operation at the slave host.

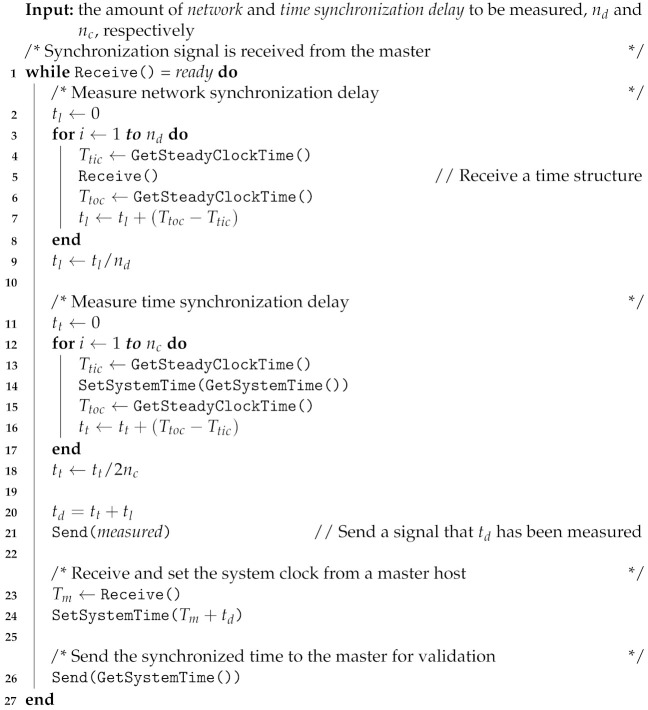




## 4. Experimental Section

### 4.1. Implementation Detail

#### 4.1.1. Capturing Studio Specification

**Figure 6:**
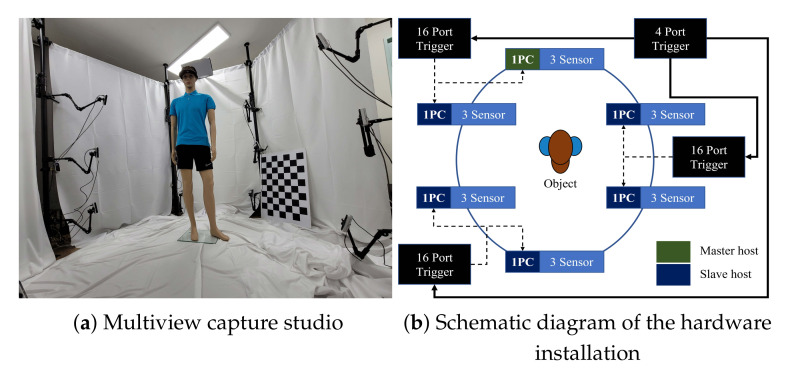
Multiview capturing system for 18 RealSense RGB-D cameras.

All the evaluations and experiments in this paper were performed using 18 Intel RealSense D455 RGB-D cameras. All cameras were connected to external triggers (*KOTRON TG-16C* and *KOTRON TG-4C*) for synchronization. In our setup, triggers were hierarchically installed. One main trigger *KOTRON TG-16C* generated periodic signals, and three subtriggers *KOTRON TG-4C* were set to bypass mode. Each subtrigger covered 6 RealSense cameras. Six desktops installed with the *Microsoft Windows 10* operating system were connected to three cameras each in order to cover the high bandwidth requirements of the cameras. Among the 6 desktops, 1 of them is selected as the master host. The schematic diagram of the hardware installation is represented in Figure 6b.

The resolution and frames per second (FPS) of the RGB-D camera were specified in pairs. We selected the depth and RGB streams according to the experimental situation. The RGB-D cameras were configured to cover 360${}^{{}^{\circ}}$ of a target object with 60${}^{{}^{\circ}}$ intervals and 3 different heights, as depicted in Figure 6a. The heights of the installed cameras were 50 cm, 100 cm, and 150 cm from the ground, respectively.

#### 4.1.2. External Trigger Synchronization

**Figure 7:**
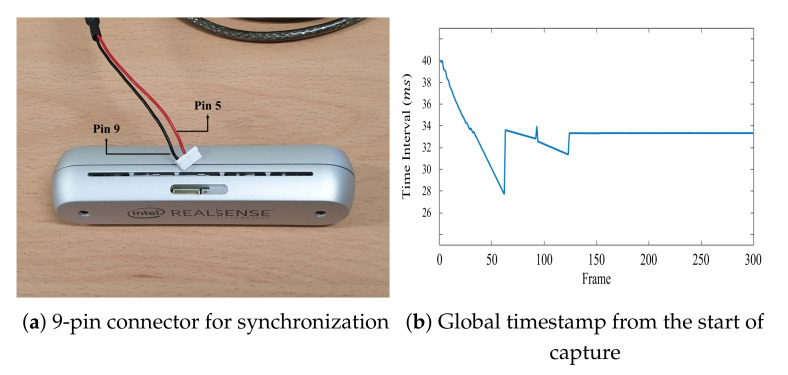
Reception of the trigger signal by the RealSense cameras.

To synchronize multiple RealSense RGB-D cameras, an external trigger was required to generate the signals at 1.8 V pulses per second (PPS). The RealSense RGB-D cameras could receive these signals through a 9-pin connector hidden inside a small latch. The sync signal was connected to pin 5, whereas pin 9 was the ground, shown in Figure 7a.

In GenLock mode, cameras do not start capturing a scene without the signal from an external trigger even though capturing is requested by the SDK. In other words, the camera’s exposure starts only when the signal from an external trigger is received. Due to this characteristic, all cameras in a multiview system can simultaneously start capturing the scene using GenLock mode. To properly use GenLock mode, an external trigger should be turned off first. Then, the start request is sent to all cameras by the SDK, and finally, the trigger is turned on and sends a signal that matches the frame rate.

Conversely, RealSense’s Slave and Full Slave modes start capturing the scene regardless of the signal’s existence. With the signal from the trigger, the cameras capture sequences synchronously. In contrast, without the signal, the cameras are unsynchronized and capture frames at a designated frame rate. Therefore, in order to synchronize the cameras in Slave and Full Slave modes, the external trigger is turned on beforehand and periodically sends the signal at a frequency that matches the frame rate. While signals are periodically sent, we can obtain the synchronized sequences by requesting cameras to start and stop capturing of the scene using the SDK.

Despite successful synchronization by GenLock, Slave, and Full Slave modes, the synchronization of cameras was prone to be unstable for the first 5 s in the RealSense camera system. Figure 7b shows the time interval of the *global* timestamps with synchronization during 10 s of recording. From Figure 7b, we can observe that the synchronization stabilized after 5 s and was able to obtain the accurate time interval of the frame rate. Hence, in order to obtain data with stable synchronization, we started recording after 5 s passes from the start of the capturing.

#### 4.1.3. Data Gathering

Each frame was captured by every camera with timestamps. Although the frames can be captured synchronously via the signal from an external trigger, the host does not receive image frames simultaneously from the RealSense cameras. It was even more difficult to determine if the images were captured simultaneously from multiple hosts. The *global* or *sensor* timestamps were used to find the simultaneously captured frames according to the proposed synchronization method. Assuming that the system times of the hosts were accurately synchronized, frames simultaneously arriving from different hosts had the same *global* timestamps. Thus, we could obtain the simultaneously captured frames by gathering each frame from all cameras that had the same *global* timestamps. The timestamps among cameras were different in practice. Therefore, we gathered frames whose timestamp differences were within a predefined threshold $T_{int}$. A value of less than a half-frame interval was sufficient for the threshold, $T_{int}$. The simultaneous frame-gathering algorithm Gathering(T,I) from the timestamp set T and image set I is presented in Algorithm 3. In Slave and Full Slave modes, the *global* timestamp set T was used to gather the frames.
**Algorithm 3:** Simultaneous frame-gathering algorithm
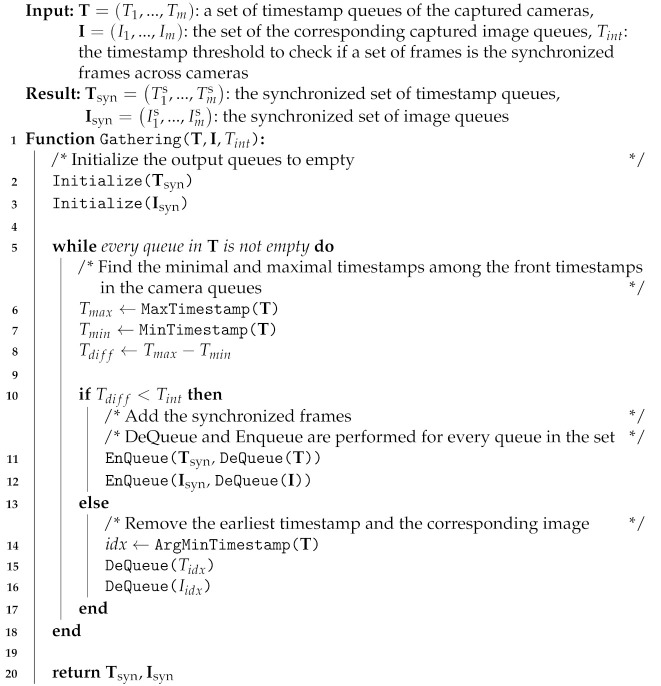


On the other hand, all the cameras are able to start capturing sequences at the same time in GenLock mode. This enabled us to use the *sensor* timestamps instead of *global* timestamps, allowing highly accurate synchronization of multiple cameras. In other words, although the devices had different *sensor* timestamps, the timestamps could be aligned with each other by subtracting each device’s timestamps by its first timestamp. Therefore, in GenLock mode, the aligned *sensor* timestamps of each camera were used to gather the frames. The aligned frames by GenLock mode were used to quantitatively evaluate if our method accurately synchronized the cameras.

### 4.2. Quantitative Evaluation

**Figure 8:**
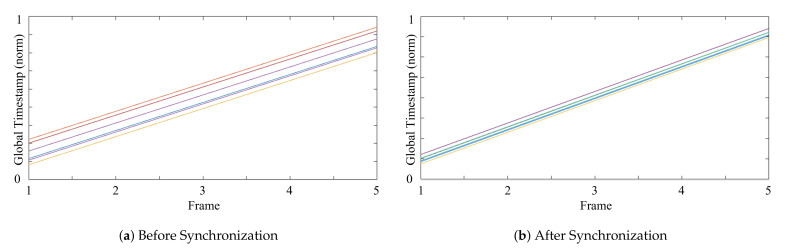
*Global* timestamps from the 18 RealSense cameras (**a**) without and (**b**) with the proposed synchronization method.

Figure 8a shows the *global* timestamps from the 18 RealSense cameras with 6 hosts. It clearly shows that the cameras connected to the same host could be correctly synchronized via the *global* timestamps, but large variations existed among the timestamps captured in different hosts. Figure 8b depicts the *global* timestamps by capturing sequences with the proposed synchronization method. It is shown that the proposed synchronization method significantly decreased the variations between hosts’ timestamps. The gathering method in Algorithm 3 matched the closest timestamps within the threshold in sequences of cameras. The lower variations in Figure 8 made it possible to gather more reliable synchronized frames.

However, the lower variations did not confirm that the cameras were synchronized correctly because the *global* timestamp was measured using the system clock of each host. To correctly evaluate the *global* timestamps of multiple hosts, the timestamps needed to be aligned with the absolute time, i.e., the ground-truth time, and then compared to each other. Therefore, in order to quantitatively evaluate the performance of our method, the ground-truth for synchronization was required. The ground-truth for synchronization was obtained by performing GenLock mode for each test.

Even though GenLock mode captures a sequence at half of the specified frame rate and synchronizes only depth cameras, it allows accurate synchronization by specifying the start time of the capturing sequences. The *sensor* timestamps of each synchronized camera could be normalized by subtracting the first *sensor* timestamp of the sequence, and with these normalized *sensor* timestamps, the frames captured by the cameras could be correctly ordered. Based on the correct ordering of the frames, the accuracy of our synchronization method could be evaluated.

We defined a metric, the average maximum delay (*AMD*), to quantitatively evaluate the synchronization of multiple RealSense cameras as:

(3) $$\begin{array}{c} AMD=\frac{1}{m}\sum_{i=1}^{m}\left(max\left(t_{g}^{i}\right)-min\left(t_{g}^{i}\right)\right), \end{array}$$

where *m* is the number of frames and $t_{g}^{i}$ is the *global* timestamp of the *i*th frame, respectively. The *AMD* measures the average difference between the maximum and minimum *global* timestamp at each frame and, as a result, can observe the accuracy of synchronization at each frame. Because the *AMD* measures the biggest difference between the cameras’ *global* timestamps at a certain frame, the *AMD* can be considered as the multiview camera system’s average maximum error. Therefore, a multiview camera system with the maximum error less than an interval between frames (i.e., 33 ms for 30 FPS) can be considered synchronized. Even though the *AMD* was useful in evaluating the performance of the multiview camera system as a whole, it was difficult to evaluate the correctness of the synchronization between the cameras.

Thus, we defined another metric, the root mean delay variation (*RMDV*), to evaluate the synchronization as:

(4) $$\begin{array}{c} RMDV=\frac{1}{m}\sum_{i=1}^{m}\sqrt{\frac{1}{n}\sum_{j=1}^{n}\left(t_{g}^{i,j}-{\hat{t}}_{g}^{i}\right)}, \end{array}$$

where *n* is the number of sensors and $t_{g}^{i,j}$ is the global timestamp of the jth sensor on the ith frame, respectively. ${\hat{t}}_{g}^{i}$ is the median value of the ith frame. The *RMDV* computes the mean variation of the delay between the cameras at each frame. In order to minimize the effect of outliers, the median of cameras’ *global* timestamp at each frame was used as a reference instead of the mean to compute the *RMDV*. The *RMDV* computes differences between the median *global timestamp* and the other cameras’ *global* timestamps and uses them to accurately measure the correctness of the synchronization.

**Figure 9:**
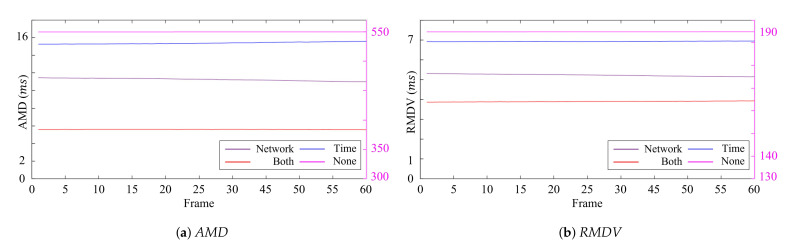
Errors of 15 FPS according to the delay for each frame (ms).

**Figure 10:**
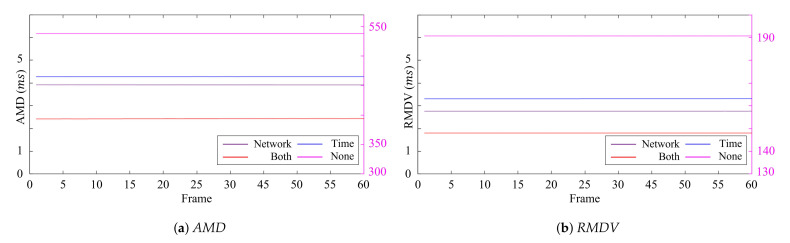
Errors of 45 FPS according to the delay for each frame (ms).

Table 2 and Table 3 show the average quantitative results over the frames of the *AMD* and *RMDV*, and the best results shown in tables are bolded for each measurement. Furthermore, the quantitative measurements for each frame are represented in Figure 9 and Figure 10. The results showed no apparent variations in the error over time regardless of the synchronization methods. This implies that the synchronization of a single host was efficiently handled with the external trigger and the *global* timestamps, and a major cause of the synchronization errors came from the synchronization error between the hosts. Our synchronization method considering *time synchronization delay* and *network synchronization delay* showed the best performance by efficiently tackling the host synchronization error, followed by applying only *network synchronization delay* or *time synchronization delay* or neither of them. From this result, we can observe that the effect of these two delays was notable.

When testing the synchronization without considering the delays, the master host was configured as a local time synchronization server, and the hosts’ system clocks were synchronized through the NTP using the time synchronization service provided by the operating system. Without the consideration of the two delays, errors in the synchronization significantly increased. When comparing the result obtained by applying only *network synchronization delay* or *time synchronization delay*, a test that only took into account the *time synchronization* caused more delay in the synchronization of the cameras than the opposite test. This was because sending and receiving data via the network communication took more time than calling the *get-time* and *set-time* operating system functions.

Table 3 shows similar results, but all tests achieved lower synchronization errors than the corresponding tests in Table 2. Because the time interval between two frames becomes smaller as the frame rate increases, as a result, delays between the cameras also decreased relatively.

### 4.3. Qualitative Evaluation

The qualitative result was obtained by capturing a sequence of a person rotating in the middle of a multiview camera system with a digital clock in his hand. The digital clock was displayed on a tablet (*SamSung Galaxy Tab S7*) with a 120 Hz display. The digital clock used in this experiment displayed the time in milliseconds in order to capture any delays between the cameras capturing the frame. Displaying time in milliseconds was appropriate for the qualitative result because the time interval between frames at 90 FPS is around 11.11 ms. While capturing a sequence, the digital clock was visible from 6 RealSense RGB-D cameras. Thus, 6 images from the RGB and depth cameras were presented for the qualitative result.

**Figure 11:**
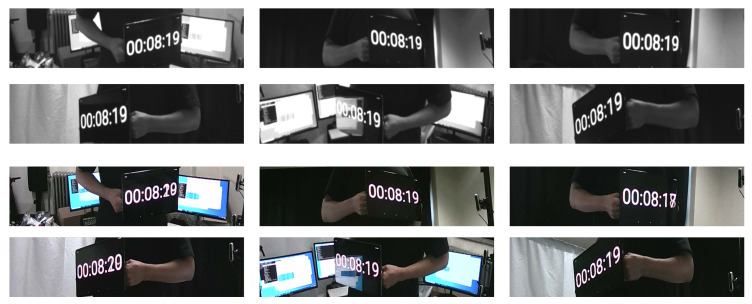
Qualitative evaluation of the RealSense GenLock synchronization.

The qualitative results obtained from GenLock mode are shown in Figure 11. As the sequences were captured with GenLock mode, the frame rate was set to half of the supported frame rate. The result showed that GenLock mode accurately captured infrared sequences synchronously. However, GenLock mode does not support synchronization between color and depth (or infrared) cameras. The inconsistent times displayed on the digital clocks show the incorrect synchronization between the color and infrared cameras.

**Figure 12:**
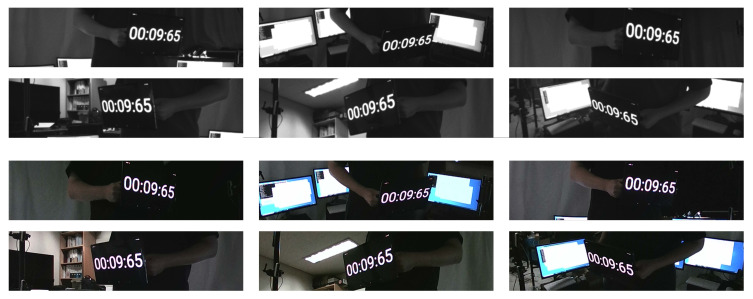
Qualitative evaluation of the proposed method. All digital clocks on the tablet show identical times.

**Figure 13:**
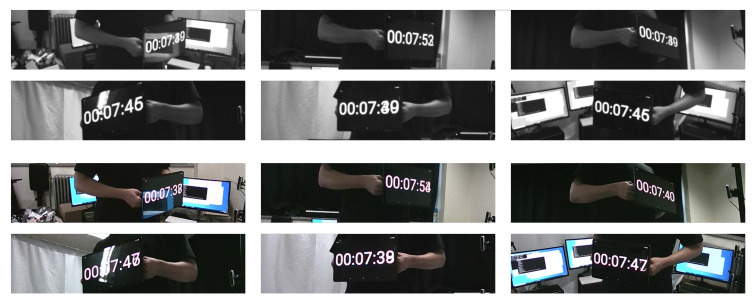
Qualitative evaluation of the NTP synchronization. The digital clocks on the tablet do not show identical times.

The qualitative results gained from the RGB-D cameras synchronized with the proposed method and the NTP are shown in Figure 12 and Figure 13. The images using the conventional time synchronization protocol showed different times displayed on the tablet. This difference in time illustrates that the cameras were not correctly synchronized. However, the frames captured by the synchronized cameras showed the identical time displayed by the digital clock. The capturing of the identical time proved that the RealSense RGB-D cameras were synchronized and were able to simultaneously capture the frames.

### 4.4. Evaluation on 3D Reconstruction

Three-dimensional reconstruction was the ultimate goal of the multiview camera system. Therefore, we evaluated the improvements of the proposed method on the reconstruction accuracy when capturing objects using multiple cameras with multiple hosts. The average $L_{2}$ reprojection error to depth maps [32] was used to quantitatively evaluate the reconstructed 3D objects from multiple views.

For the 3D reconstruction from the multiview depths, we first calibrated the depth sensors with the standard multicamera calibration method using a checkerboard [33]. Then, using the extrinsic calibrated depth camera, 3D points in the local camera coordinates could be integrated into the global coordinates. The Poisson reconstruction method [34] was applied to the integrated point set to construct a 3D mesh of the target object.

The reprojection error was computed by projecting the vertices of the mesh to every depth map to match the corresponding points of the depth map to the 3D vertices. When calculating the reprojection error, the 3D vertices invisible to each depth camera were excluded, which could be determined by the Z-buffer test [35]. Once the correspondences between the mesh vertices and depth points were matched, the $L_{2}$ distance errors in the global coordinates were calculated and averaged.

Table 4 summarizes the reprojection errors without and with the proposed synchronization method when capturing the objects with Slave and Full Slave modes. When capturing the sequence without the proposed synchronization, the standard NTP synchronization scheme was used. The result demonstrated that the proposed method was significantly beneficial in obtaining accurate and reliable reconstruction results from the multiview camera system. The qualitative comparison is described in Figure 1. The mesh reconstructed using the synchronization scheme without the proposed method showed notable artifacts arising due to the misalignment among the multiview depths. This misalignment was caused by inaccurate synchronization between hosts. In contrast, the proposed method enabled robust reconstruction from multiple cameras with multiple hosts by efficiently addressing the synchronization problem.

### 4.5. Discussion

The proposed method resolved the problems in the Slave and Full Slave synchronization modes of the RealSense devices by synchronizing multiple hosts’ times. In our experiments, the RealSense cameras were connected to the motherboard USB 3.0 interfaces of the hosts for communication without using an additional PCI-E extension card. It has been reported that the number of RealSense sensors that a host can support is up to three [18] without using any extension card. Thus, we used six hosts to support eighteen cameras for our experiments. However, if utilizing extension cards for the USB interface, a host can support a wider bandwidth and stably connect to more cameras, as long as the number of cameras connected to a host does not exceed the hardware limitation. In this case, the multiview camera system can be accurately synchronized using a single host without the proposed method.

Furthermore, although the GenLock synchronization mode of the RealSense cameras drops the frame rate by half and cannot synchronize RGB cameras simultaneously with cameras, GenLock mode can be used for other cameras to accurately synchronize multiple RGB-D cameras without the frame rate loss, when all cameras start capturing at the same time. In other words, other depth cameras can be easily synchronized without the proposed synchronization method if using GenLock mode.

However, in many practical applications, it is difficult to specify the starting point of capturing for each camera. As a result, the typical GenLock synchronization scheme cannot be used, but the proposed method can still be used to accurately synchronize multiple cameras with GenLock mode. In addition, our method does not limit the number of devices that can be synchronized by linearly increasing the number of hosts, allowing the multiview camera system to be largely scalable.

## 5. Conclusions

In this paper, we proposed a novel synchronization method for synchronizing RealSense RGB-D cameras in a multiview camera system. The proposed method tackles the limitation of RealSense camera synchronization, where multiple camera synchronizations using multiple hosts are possible with dropping the frame rate and depth cameras only. As the number of RealSense cameras to be used increases, more hosts are required to support a multiview camera system. To synchronize the RealSense cameras connected to different hosts, the hosts’ system clocks were synchronized in order to correctly regress the *global* timestamps of all cameras. The system clocks were synchronized by estimating the delay occurring while synchronization takes place. The estimation of the delay minimized the offset between the master host’s system clock and the slave hosts’ system clocks. The result quantitatively and qualitatively showed that our method reduces the synchronization error between sensors significantly, enabling simultaneous capturing by numerous RealSense cameras without dropping the frame rate. We expect that our method will be helpful in various applications of computer vision related to 3D reconstruction [9,32].

## Figures and Tables

**Table 1:** Specifications of commercial RGB-D cameras.

Features	Kinect Azure	Astra S+	Astra S
Technology	Time-of-flight	Structured light	Structured light
RGB Res. (30FPS)	3840 × 2160	1920× 1080	1280× 720
Depth Res. (30 FPS)	640× 576	640× 480	640× 480
Depth Res. (90 FPS)	*Not Supported*	*Not Supported*	*Not Supported*
Size (mm)	103 × 39 × 126	149 × 28 × 29	165 × 30 × 40
Power Supply	DC + USB 3.0	USB 3.0 Type-C	USB 2.0
Multi. Capability	△	×	×
**Features**	**RealSense D455**	**RealSense D435**	**RealSense L515**
Technology	Active stereo	Active stereo	LiDAR
RGB Res. (30 FPS)	1280 × 800	1920 × 1080	1920 × 1080
Depth Res. (30 FPS)	1280× 720	1280× 720	1024 × 768
Depth Res. (90 FPS)	848 × 480	848 × 480	*Not Supported*
Size (mm)	124 × 26 × 29	90 × 25 × 25	61 × 61 × 26
Power Supply	USB 3.0 Type-C	USB 3.0 Type-C	USB 3.0 Type-C
Multi. Capability	◯	◯	×

**Table 2:** Errors of 15 FPS according to delay (ms).

Added Delay	*AMD*	*RMDV*
*Network synchronization*	11.1648	5.2394
*Time synchronization*	15.3963	6.9118
Both delays	**5.8539**	**3.8868**
None	547.5033	194.8521

**Table 3:** Errors of 45 FPS according to delay (ms).

Added Delay	*AMD*	*RMDV*
*Network synchronization*	3.9161	2.7667
* Time synchronization*	4.2783	3.3137
Both	**2.4380**	**1.8044**
None	538.0590	190.7111

**Table 4:** Reprojection errors (cm).

Added Delay	Mean	±Std.
*RealSense Sync.*	9.7030	12.1391
*Proposed Sync.*	**2.3942**	**1.7967**

## Data Availability

Not applicable.

## Abbreviations

The following abbreviations are used in this manuscript:

RGB-D	Red, green, blue-depth
3D	Three-dimensional
SDK	Software development kit
USB	Universal serial bus
GenLock	Generator locking
ToF	Time-of-flight
LiDAR	Light detection and ranging
IR	Infrared
OS	Operating system
PPS	Pulses per second
AMD	Average maximum delay
RMDV	Root mean delay variation
FPS	Frames per second
NTP	Network Time Protocol
